# Dentists' Practices and Attitudes Toward Using Personal Protection Equipment and Associated Drawbacks and Cost Implications During the COVID-19 Pandemic

**DOI:** 10.3389/fpubh.2021.770164

**Published:** 2021-11-18

**Authors:** Nibras H. Chasib, Muhanad L. Alshami, Sarhang S. Gul, Hayder R. Abdulbaqi, Ali A. Abdulkareem, Salah A. Al-Khdairy

**Affiliations:** ^1^College of Dentistry, University of Baghdad, Baghdad, Iraq; ^2^Department of Dentistry, Dijlah University College, Baghdad, Iraq; ^3^College of Dentistry, University of Sulaimani, Sulaymaniyah, Iraq; ^4^Mydentist Bridgend, Bridgend, United Kingdom

**Keywords:** personal protection equipment, dentistry, COVID-19, knowledge, cost

## Abstract

**Objectives:** During the COVID-19 pandemic, dentists have had to work under stressful conditions due to the nature of their work. Personal protection equipment (PPE) has become mandatory for work in the dentistry field. This study aimed to examine dentists' practices and attitudes regarding the use of PPE and the associated drawbacks and cost implications during the pandemic.

**Methods:** A questionnaire-based survey was used and was divided into five sections dedicated to collect demographic variables and to examine the dentists' practices, attitudes toward PPE, drawbacks, and cost of using PPE. Mann-Whitney U and Kruskal-Wallis tests were used to compare different sections of the questionnaire and linear regression was used to determine the predictors of the dentists' practices and attitudes toward PPE.

**Results:** The mean of practices regarding use of PPE was 5.41 ± 1.71 (median 6), attitude toward using PPE scored 2.26 ± 0.90 (median 2), while the means of drawbacks and cost recorded equal scores of 5.22 ± 1.24 (median 5) and 1.68 ± 0.74 (median 2), respectively. The recently graduated dentists, those with postgraduate degrees and those working in the private sector demonstrated higher levels of practices on PPE than their counterparts. Regression analysis revealed that practices of PPE can be predicted on the basis of qualifications and work place, whereas attitude toward PPE is significantly influenced by qualification only.

**Conclusions:** The study participants demonstrated satisfactory levels of practices and positive attitudes toward PPE. While complaints from using PPE and their cost were moderately affected.

## Introduction

Toward the end of 2019, the whole world was faced with a new and dangerous challenge about which there was very little information and which was later to become known as the COVID-19 virus. Early reports and warnings from the World Health Organization (WHO) about this virus were released in the weekly updates on January 12 when China shared the genetic sequence of the virus (1). One year later, on 29/12/2020, the WHO reported 79 million reported cases and over 1.7 million deaths (2).

The typically reported symptoms of COVID-19 include fever, cough, fatigue, anorexia, shortness of breath and myalgia in addition to other reported symptoms such as loss of smell and taste (3, 4). However, many COVID-19 cases are now appearing that have less typical symptoms or are asymptomatic (5). People in the latter category pose a particular risk to dental staff as they may unknowingly attend their dental appointment while infected with COVID-19 (6, 7).

Dental staff are also at increased risk due to their proximity to the patient's nose and mouth while they work (4). In addition, the aerosols generating procedures (AGPs) cause emission of airborne droplets from the patient's mouth into the surrounding environment, which is one of the main routes of transmission of COVID-19 (8). The aerosols in a COVID-19 positive patient will likely contain a high viral load, further increasing the risk of transmission to the dental staff during a single session (9). Dental staff around the world were forced to cease or highly restrict the treatment that they offered as the COVID-19 outbreak became a pandemic (8). Research into how to safely return to dental treatment was now the priority and resulted in the introduction of new protocols for use with enhanced personal protection equipment (PPE) (10). Implementation of AGPs now requires the use of a respirator mask, most commonly the FFP2 (N95) or FFP3 or equivalent masks (10). However, their use comes at greatly increased financial cost, with the WHO reporting on 3rd March 2020 that the price of surgical masks had multiplied by 6-fold and N95 respirators by 3-fold and the cost of gowns had doubled (1). Further, the United Kingdom has also implemented a “fallow time” to allow aerosols to settle following provision of AGPs and then performance of cleaning to help decrease risk of transmission.

The new protocols have succeeded in decreasing the risk of transmission in the dental practice setting (11). However, certain negative factors need to be addressed, such as increased anxiety among dentists and dental staff (12) and the physical difficulties associated with wearing enhanced PPE (13). Additionally, there is the time loss due to fallow time and the extra time needed for donning and doffing (14, 15). All of these factors, along with the increased cost of enhanced PPE and mitigation factors, contribute to decreasing the number of patients that can be seen in a day and therefore to a decrease in earnings (16, 17). In Iraq, as in other countries, there was an initial lack of information and evidence-based guidelines regarding protection and prevention of COVID-19 transmission due to its novelty. The aim of this study was to investigate the dentists' practices and attitudes regarding PPE and the drawbacks of using PPE in dental practice.

## Methods

### Study Design and Population

This study was a cross-sectional survey conducted among Iraqi dentists between December 2020 and January 2021. An electronic version of the questionnaire was randomly emailed to the dentists after obtaining the relevant ethical approval in accordance to the Helsinki declaration for human research from the College of Dentistry, University of Sulaimani (ethical approval number: 17/21).

Registered dentists who agreed to respond to the questionnaire were included in the study. Dentists who returned incomplete questionnaires and those who declined to participate were excluded. The number of registered dentists was officially obtained from the Iraqi Dental Association (IDA). The questionnaire was left open initially for 2 weeks, then a reminder was sent and it was kept open for an extra week before it was closed and data were retrieved.

### Sample Size Calculation

The total number of registered Iraqi dentists was used to estimate the sample size. Iraqi Dental Association has officially provided the authors with required information about total number of dentists (6,463) and their distribution across Iraqi governates. Accordingly, sample size required to reject null hypothesis [at 5% error margin and 95% confidence interval (CI)] was calculated as follows:


$$\begin{array}{l} \text{Sample size = }(\text{distribution of 0}.\text{5})\text{/}\left(\left(\text{error}\;\text{margin}\%\text{/CI}\right)\right)\\ \text{Where CI = 1}.\text{96 }(\text{for CI of 95}\%),\text{ error margin =}\;\text{0}.\text{05}.\\ \text{True sample = }(\text{sample size }\times \text{ population})\text{/}(\text{sample size}\\ \;\text{+ population -}\;\text{1}) \end{array}$$

Therefore, the required sample size was 363 dentists which was rounded up to 400. To avoid a potential dropout estimated by a previous pilot study, the number of distributed questionnaires was doubled i.e., 800.

### Questionnaire Elements and Scoring

The questionnaire (Table 1) was adapted from previous studies (17–19) and divided into 5 sections, the first of which concerned demographic variables. The second section was intended to assess the dentists' practices about using PPE (from question #1 to #9). The third section (from question #10 to #13) was dedicated to evaluating the attitude toward PPE. Sections 4 (from question #14 to #20) and 5 (from question #21 to #23) were designed to assess the drawbacks and cost implications of PPE, respectively.

**Table 1:** Elements of the questionnaire.

- Gender					
	Male	Female			
- Age:					
- Qualification					
	BDS	HDD	MSc	PhD	
- Graduation year?					
- Work place					
	Private	Governmental	Both		
When did you resume treating patients following easing of COVID-19 pandemic restrictions?			≤ 3 months	>3 months	Not resumed my work yet
How many patients (on average) have you seen per day since the COVID-19 pandemic started?					
Response for all questions is Yes or No					
1- Do you use a surgical mask for all dental procedures?					
2- Do you use respirators (e.g., N95) when indicated?					
3- Do you use a face shield?					
4- Do you use garments (gown, tunic/scrubs) for aerosols generating procedures (AGP)?					
5- Do you use garments for non-AGP procedures?					
6- Did you do fit test before wearing your mask?					
7- Do you use a head cover?					
8- Do you think that mishandling PPE is a potential source of COVID-19 transmission?					
9- Are you using other protection equipment (e.g., Ventilators, Air filtration systems, Physical barriers/screens)?					
10- Do you feel safe wearing your PPE?					
11- Is it very important to wear the full PPE?					
12- Using PPE has no impact on the communication with the patient?					
13- Are you satisfied with the extra cost of the PPE?					
14- Do you feel fatigue/tired during or at the end of the dental treatment?					
15- Is your PPE impacting your clinical time?					
16- Do you experience increases in sweating/body temperature while wearing your PPE?					
17- Is it hard to put on (don) the full PPE?					
18- Is it hard to remove (doff) the full PPE?					
19- Is it hard to hear/see around you while wearing the full PPE?					
20- Is it hard to make decisions while wearing the full PPE?					
21- Did your budget for purchasing PPE increase after the COVID-19 pandemic?					
22- Did you increase the fees for treatments to compensate for PPE cost?					
23- During the pandemic, the PPE was not cost effective?					

*The bold number are significant, the bold word are separation between sections of questionnaire*.

Questions in the practices, attitude, drawbacks and the cost sections were closed-ended and required a “Yes” or “No” response. Each “Yes” response was scored as “1” while “No” response received score “0.” For all these sections, the mean of answers per participant was calculated and used for the analysis. Percentage responses for each question were used to express different aspects of each section.

### Statistical Analysis

Frequency, percent, mean and standard deviation were used as descriptive statistics for demographic variables. These variables were dichotomized according to sex (male and female), qualification (BDS or other higher degrees), or depending on the median value for graduation year (<2013 or ≥2013) and when resuming work ( ≤ 3 months or >3 months). While work place was categorized as governmental, private, and both. Mann-Whitney U test and Kruskal-Wallis test were used to compare continuous data of different sections of the questionnaire. Multiple linear regression analysis was used to determine predictors of the dentists' practices and attitudes toward PPE. Significance level was set at *p* < 0.05. All statistical analyses were performed by using SPSS (version 25, IBM, USA).

## Results

A total of 333 dentists responded fully to the questionnaire representing 41.6% response rate, with average age of 33.8 ± 7.7 years old. Distribution of the study population according to the study variables is illustrated in Table 2. Individual responses for each question are illustrated in Table 3.

**Table 2:** Demographic and general practice characteristics of dentists since COVID-19 outbreak (*N* = 333).

**Variable**	**Number (*n*)**	**Percent (%)**
Age (mean ± SD)	33.83 ± 7.68	
Patients seen/day since COVID-19 (mean ± SD)	7.14 ± 9.92	
**Sex**		
Male	185	55.6
Female	148	44.4
**Qualification**		
BDS	224	67.3
HDD or MSc or PhD	109	32.7
**(BDS) Graduation year**		
<2013	157	47.1
≥2013	176	52.9
**Work place**		
Governmental	92	27.6
Private	83	24.9
Both	158	47.4
**Resuming work after COVID-19 pandemic restriction break**		
≤ 3 months	173	51.9
>3 months	160	48.1

**Table 3:** Responses (score 1) per question for each section (*N* =333).

**Practices about PPE**	**Responses^**§**^**	**Attitude toward PPE**	**Responses^**§**^**
Q1	169 (50.8)	Q10	216 (64.9)
Q2	205 (61.6)	Q11	286 (85.9)
Q3	266 (79.9)	Q12	149 (44.7)
Q4	271 (81.4)	Q13	100 (30.0)
Q5	213 (64.0)	**Drawbacks of PPE**	
Q6	117 (35.1)	Q14	309 (92.8)
Q7	187 (56.2)	Q15	250 (75.1)
Q8	293 (88.0)	Q16	303 (91.0)
Q9	81 (24.3)	Q17	225 (67.6)
**Cost of PPE**		Q18	231 (69.4)
Q21	276 (82.9)	Q19	178 (53.5)
Q22	65 (19.5)	Q20	243 (73.0)
Q23	220 (66.1)		

§ *Frequency (percent)*.

Practices of using PPE according to different situations was significantly higher among postgraduate degree holders than among general practitioners, and among recently graduated dentists compared to those who graduated before 2013 (Table 4). In addition, dentists working in the governmental sector demonstrated significantly lower practices than those working in the private or private/governmental sector (Table 4). No significant differences were observed in attitude toward PPE according to the different variables except in the case of postgraduate qualifications, which showed a statistically significant association with a more positive attitude toward PPE when compared with Bachelor degree. Similarly, the demographical variables were found to have no significant effect on reported drawbacks of PPE or its cost (Table 4). Overall, the mean of practices of using PPE was 5.41 ± 1.71 (median 6), attitude toward using PPE scored 2.26 ± 0.90 (median 2), while the means of drawbacks and cost recorded equal scores of 5.22 ± 1.24 (median 5) and 1.68 ± 0.74 (median 2), respectively (Table 4).

**Table 4:** Responses on practice and attitude toward using PPE, the associated drawbacks and cost implications according to study variables.

**Variable**		**Practice of using PPE**	**Attitude toward PPE**	**Drawbacks of PPE**	**Cost of PPE**
**Sex**					
Male^§^		5.45 ± 1.69	2.24 ± 0.93	5.21 ± 1.26	1.65 ± 0.73
Female		5.35 ± 1.75	2.27 ± 0.87	5.23 ± 1.23	1.71 ± 0.75
*p*-value^*^		0.771	0.752	0.816	0.585
**Qualification**					
BDS^§^		5.18 ± 1.68	2.18 ± 0.88	5.21 ± 1.28	1.68 ± 0.77
HDD or MSc or PhD		5.88 ± 1.70	2.40 ± 0.93	5.24 ± 1.18	1.69 ± 0.68
*p*-value^*^		**0.0001**	**0.033**	0.331	0.761
**(BDS) Graduation year**					
<2013^§^		5.66 ± 1.84	2.32 ± 0.91	5.23 ± 1.17	1.71 ± 0.72
≥2013		5.18 ± 1.56	2.19 ± 0.89	5.21 ± 1.31	1.67 ± 0.75
*p*-value^*^		**0.004**	0.175	0.942	0.561
**Work place**					
Governmental^§^		4.90 ± 1.55	2.23 ± 0.85	5.28 ± 1.14	1.54 ± 0.77
Private		5.68 ± 1.89	2.22 ± 0.97	5.12 ± 1.31	1.78 ± 0.74
Both		5.56 ± 1.66	2.27 ± 0.90	5.24 ± 1.27	1.71 ± 0.70
*p*-value^**^		**0.004**	0.867	0.722	0.178
**Resuming work after restriction easing**					
≤ 3 months^§^		5.35 ± 1.75	2.26 ± 0.90	5.27 ± 1.20	1.75 ± 0.75
>3 months		5.47 ± 1.68	2.25 ± 0.91	5.16 ± 1.29	1.61 ± 0.72
*p*-value^*^		0.933	0.596	0.507	0.059
Overall	Mean ± SD	5.41 ± 1.71	2.26 ± 0.90	5.22 ± 1.24	1.68 ± 0.74
	Median	6	2	5	2

§ *Reference variable*.

* *Comparison by Mann-Whitney U test, with significance level at 0.05*.

** *Comparison by Kruskal-Wallis test, with significance level at 0.05; practices of using PPE differs between governmental and private work place (adjusted p-value of 0.018) and between governmental and both work places (adjusted p-value of 0.006). The bold numbers are significant, the bolds word are the separation between sections of questionnaire*.

Regression analysis models indicated that predictors of practices of using PPE were qualifications and work place, while dentist's qualification was the only predictor of attitude (Table 5).

**Table 5:** Predictors for practices of using PPE and attitude toward PPE (multiple linear regression).

**Dependent variables^**§**^**	**Predictors**	**Unstandardized coefficients**	**R square**	**Constant**	***p*-value**
Practices of using PPE	Qualification	0.072	0.053	0.543	0.001
	Work place	0.029			0.017
Attitude toward PPE	Qualification	0.055	0.013	0.546	0.037

§ *No contributors were detected for changes in reporting drawbacks of PPE and cost of PPE*.

## Discussion

While PPE plays a valuable role in providing protection from infection, lack of practices about its use reduces its benefits and can give a false impression of safety that may increase the infection risk. This study aimed to investigate the practices and attitude of dentists toward PPE together with associated drawbacks and cost aspects. Significant differences in practices about using PPE were identified among participants in the current study in relation to qualification, graduation year, and work place. Meanwhile, attitude toward using PPE was mainly associated with qualification.

Generally, dental staff are aware of and familiar with using conventional PPE; however, this could not be the case with the new guidelines for using PPE introduced as a consequence of the recent pandemic (20). In particular, the new rules on protection that require most of the body to be covered present problems in achieving correct application (20). This issue was highlighted in the current survey as only 35% of the dentists reported checking the fitting of PPE, an additional step that most health workers usually neglect. The requirement to use additional items has led to doffing and donning PPE becoming a more difficult and time-consuming process (20). This finding is confirmed by the results of this study, with 67–69% of the dentists considering doffing and donning to be tedious and hard processes. Furthermore, the introduction of ventilators, high-suction devices and other extra equipment to maximize safety during dental work was not well-perceived by the participants, possibly due either to lack of practices about the advantages or concern over the high cost. Overall, the dentists showed satisfactory levels of practices about the use and indications of different PPE. Although only 30% of the dentists were satisfied with the increase in cost of PPE during the pandemic, about 85% of them were aware of the importance of PPE. This reflected a generally positive attitude among the participants toward the use of PPE.

While protection from the virus is necessarily a priority, the comfort and satisfaction of the dentists are also prerequisites for high quality treatment (17). Health workers using PPE for long periods have reported different complications including fatigue, dehydration, and headaches (21). These adverse effects were further complicated by fear of infection which added further stress that significantly impacted their decision making and quality of treatment (21). These factors were observed in our study as fear of infection was expressed by about 35% of the participants, even though they were using proper PPE. Further, participants also reported that using PPE had a negative impact on their decision making and communication with their patients.

The majority of PPE-related symptoms are mainly attributed to heat-related illness which is predisposed by many risk factors, such as physical exertion, heat and humidity accumulation, and dehydration (22). In addition, physical effects of wearing PPE such as facial bruises have been reported by other health workers (23). In responses to a questionnaire-based survey conducted in India, all participants reported excessive sweating as a main problem of using PPE (24). The same study also indicated a range of other PPE-related problems including reduced vision due to fogging, suffocation, shortness of breath, headache, pressure marks, and occasional skin allergies/dermatitis (25). These results were consistent with the current study in which over 90% of the participants indicated fatigue and excessive sweating/increased body temperature as the main adverse effects of using PPE.

The majority of participants reported that their budget for purchasing PPE increased after the pandemic. However, over 60% of them thought that PPE was cost-effective and <20% of the dentists actually increased the treatment cost to compensate for the change in PPE cost. The escalation of public panic, especially in the early months following the onset of the COVID-19 crisis, led to demand for PPE exceeding the production capacity. The resulting depletion of PPE stocks then caused a significant increase in the prices. Later, the supply problem was alleviated by locating alternative sources and increasing production, but the price of PPE remained relatively high. Worldwide, the need to obtain extra PPE items increased the financial burden on the health authorities and health workers. For instance, the Aneurin Bevan Health Board Oral and Maxillofacial Unit, UK reported additional expenditure of £32,292 on purchasing PPE to meet the requirements of the new protection guidelines (25). Moreover, according to a tracking poll conducted by the ADA Health Policy Institute, one-third of the participating dentists reported a triple increase in their PPE costs (26). Further, the cost of surgical masks increased by up to 10-fold post-COVID-19 as compared to pre-pandemic (27).

The analysis showed that higher degree holders and those who had graduated in the after 2013 had greater practices about PPE. This indicates that educational achievement is a predictor of practices and positive attitude toward PPE. Recently graduated dentists could also have acquired practices via social media and other online sources which could be less attractive information sources for older graduates. Interestingly, those working in the private sector had better practices than their peers in governmental centers. This could be attributed to dentists working in the private sector having more responsibility and therefore being more concerned regarding the safety of the clinics on which their livelihoods depend. Furthermore, providing safe and good practices in the private sector could be related to the fact that patients seeking dental treatment expect better practice as the cost is high and dentists working in the private sector are aware of this expectation and try to keep up to date, otherwise, they might affect their reputation in the future. On the other hand, dentists working in governmental centers work in shifts that minimize their contact with the patients and use PPE provided according to the guidelines approved by the health authorities.

There is no doubt about the vital role of PPE in decreasing the transmission of the virus and saving lives. Meanwhile, problems associated with the tolerability and stress of wearing PPE were found to be greatly reduced by using Powered Air Purifying Respirators (28). In addition, using breathable PPE that meets high protection standards will have a positive impact on the quality of the treatment and the dentist's comfort during the work.

These dentists' responses cannot be regarded as an accurate reflection of their actual practice or behavior in real life, which represents a limitation of this type of study. Furthermore, the increased demand for PPE led to a lack of quality control due to the market being flooded with different brands from different origins, which could have affected the attitudes of the dentists toward PPE. Future research is encouraged on duties and responsibilities of dentists working in private dental practices in comparison to those working in governmental canters and impact of previous years of experience on practices and attitude of dentists toward PPE.

## Conclusions

The current survey indicated that the dentists had satisfactory levels of practices and positive attitudes toward using PPE. Additionally, levels of complaints about using PPE together increased cost of purchasing PPE were moderately affected.

## Data Availability Statement

The raw data supporting the conclusions of this article will be made available by the authors, without undue reservation.

## Ethics Statement

Ethical approval in accordance with the Helsinki declaration for human research was given by the College of Dentistry, University of Sulaimani (ethical approval number: 17/21). Written informed consent for participation was not required for this study in accordance with the national legislation and the institutional requirements.

## Author Contributions

NC and HA participated in the design and conception of the study, first draft of the manuscript and its coordination. MA and SA-K participated in acquisition of data and first draft of the manuscript. SG and AA carried out statistical analysis and edited the final draft of the manuscript. All authors contributed to the article and approved the submitted version.

## Conflict of Interest

The authors declare that the research was conducted in the absence of any commercial or financial relationships that could be construed as a potential conflict of interest.

## Publisher's Note

All claims expressed in this article are solely those of the authors and do not necessarily represent those of their affiliated organizations, or those of the publisher, the editors and the reviewers. Any product that may be evaluated in this article, or claim that may be made by its manufacturer, is not guaranteed or endorsed by the publisher.
